# 

**Published:** 1889-10-26

**Authors:** 


No. 161, Vol. VII.—October 26th, 1889.

[Registered at the General Post Office as a Newspaper for transmission in the United Kingdom and Abroad.

Per Annum, 6s. 6d., post free.

Monthly Parts, 6d.; post free 7½d.

Ditto per Annum, 7s. 6d., post free.

